# Current GMP standards for the production of vaccines and antibodies: An overview

**DOI:** 10.3389/fpubh.2022.1021905

**Published:** 2022-11-03

**Authors:** Consuelo E. Covarrubias, Thomas A. Rivera, Catalina A. Soto, Trevor Deeks, Alexis M. Kalergis

**Affiliations:** ^1^Millenium Institute on Immunology and Immunotherapy, Departamento de Genética Molecular y Microbiología, Facultad de Ciencias Biológicas, Pontificia Universidad Católica de Chile, Santiago, Chile; ^2^Deeks Pharmaceutical Consulting Services, Rockville, MD, United States; ^3^Departamento de Endocrinología, Facultad de Medicina, Pontificia Universidad Católica de Chile, Santiago, Chile

**Keywords:** good manufacturing practices (GMP), world health organization (WHO), vaccines, antibodies, Bacillus Calmette-Guerin (BCG)

## Abstract

The manufacture of pharmaceutical products made under good manufacturing practices (GMP) must comply with the guidelines of national regulatory bodies based on international or regional compendia. The existence of this type of regulation allows pharmaceutical laboratories to count on the standardization of high-quality production processes, obtaining a safe product for human use, with a positive impact on public health. In addition, the COVID-19 pandemic highlights the importance of having more and better-distributed manufacturing plants, emphasizing regions such as Latin America. This review shows the most important GMP standards in the world and, in particular, their relevance in the production of vaccines and antibodies.

## Introduction

Every pharmaceutical product administered to humans or animals must be manufactured under good manufacturing practices (GMP) standards. The guide is divided into three parts. The first specifies the principles for the manufacture of medicinal products, the second specifies the basic requirements for active substances used as starting materials, and the third clarifies all the documentation for regulatory certification. Worldwide, each country uses a GMP regulation based on the WHO guide or the EU guide. The first GMP guide was published in 1971 in the UK (the Orange Guide) (1) and applies to medical products for human and veterinary use. It is currently undergoing an update for sterile pharmaceuticals such as vaccines and monoclonal antibodies, which must be manufactured in aseptic conditions. This type of manufacturing is the most challenging and risky process in the industry.

The Severe Acute Respiratory Syndrome Coronavirus 2 (SARS-CoV-2) pandemic highlighted the importance of vaccines to control the Coronavirus Disease 2019 (COVID-19) consequences. Most of these GMP facilities are in the EU, USA, China, and European countries. To facilitate the access of these types of products to Latin American countries, the expansion of production capacities and installation of new GMP production plants is essential.

In this article, we compare the differences between the WHO and EU GMP guides, the principles that regulate the production of vaccines and monoclonal antibodies, and the impact of the GMP facilities on public health.

## Good manufacturing practice (GMP)

The GMP normative is defined to facilitate the barriers to trade in medicinal products, promote uniformity in licensing decisions, and ensure the maintenance of high-quality assurance standards in developing, manufacturing, and controlling medicinal products. A system of marketing authorizations ensures that all medical products are assessed by a competent authority to ensure compliance with the requirements of safety, quality, and efficacy.

### History of GMP regulations

The importance of GMP standards and their contribution to all consumers can be traced back to tragic circumstances that affected many human lives in 1906 due to deficient standards in the meat industry (2). Publications and public outcry prompted the US Pure Food and Drug Act passage that same year (3). This law prohibited the transportation of illegal food and drugs under the penalty of confiscating the products and prohibition of marketing. In addition, it mandated the sale of medicines that met USP and National Formulary standards, which were legibly indicated on the label (4).

After nearly 107 children died in 1937 from using sulfanilamide elixir, which contained a chemical used as an antifreeze, the Federal Food, Drug, and Cosmetic Act was passed in 1938 in response to the tragedy (5). This Act required that manufacturers must demonstrate that a drug was safe before marketing it, and therapeutic devices were regulated for the first time. Subsequently, in 1962, the Medicines Amendments were unanimously adopted in the wake of the thalidomide incident. These Amendments strengthened the control of prescription, new, and investigational drugs. In addition, efficacy demonstration was required before market release, supported by reports showing possible adverse reactions (6). Moreover, in the case of investigational use, manufacturers were required to inform participants of the characteristics of the drug in use, together with the potential harms, and to obtain the patient's consent before testing it on them. In addition, this law gave the FDA the authority to regulate the advertising of prescription drugs. Finally, in 1978, GMPs for drugs and medical devices were developed. They were intended to help ensure the safety and efficacy of all products, and in 1979 they became law (6).

### International regulatory cooperation

The first WHO draft text was adopted in 1968, and 1 year later, the World Health Assembly recommended the first version of the WHO Certification Scheme on the quality of pharmaceutical products (7). The guidelines were expanded from 1989 until 1990, and today more than 100 countries have incorporated the WHO GMP guide into their national medicine laws, such as Chile, which has its guideline based on the WHO GMP Guide (8–10), and Canada, which also developed a particular guideline based on the EU guide (11).

**Figure 1:**
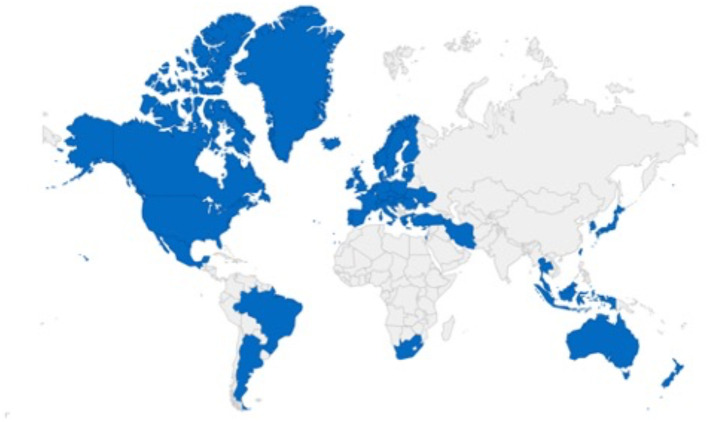
Pharmaceutical Inspection Co-operation Scheme (PIC/S), the representative countries included in the Convention (9). The blue sections indicate the locations of representatives participating in the PIC/S GMP convention, and the gray areas indicate countries that do not participate in the PIC/S GMP convention and are therefore governed by other GMP regulations.

The Pharmaceutical Inspection Co-operation Scheme (PIC/S) is a non-binding, informal cooperative arrangement between Regulatory Authorities in Good Manufacturing Practice of medical products for human or veterinary use. Countries that follow PIC/S guidelines include Australia, New Zealand, South Africa, Argentina, Brazil, Mexico, Australia, Turkey, Japan, Belgium, Denmark, Estonia, Finland, France, Germany, Hong Kong, and others. Figure 1 shows the participating authorities in the world at present (12–15).

The EU GMP guidelines and all the annexes associated with or guide qualification and validation are the same regulations and policies that apply in the PIC/S countries, except for references to European legislation. Also, Annex 16 relating to the Qualified Person is either not applicable or is interpreted slightly differently (12, 13).

EU GMP guidance began in the 1970s, following a series of disastrous incidents in the 1960s, e.g., the thalidomide drug incident mentioned above. These incidents led to the publication of the Clothier Report and, subsequently, in 1971, the first GMP regulations in the UK, better known as the Orange Guide (1). Since then, the text has continued to be modified. In 1993, they became part of the “Standards and Guidance for Manufacturers and Distributors of Pharmaceuticals”, which regulate and guide manufacturing practices and include the EU GMP guidelines. The first version of the Guide did not mention the validation process in manufacturing pharmaceutical products, analytical activities, or clinical studies. Validation is the demonstration of process performance. The terms “commissioning and testing” were used in the 1970s to refer to the “qualification” of autoclaves, sterilization ovens, and clean rooms (13). In 1970, grades A, B, C, and D were defined for pharmaceutical cleanrooms. The fundamental principles have not changed over time. However, the EU GMP did not mention the validation process then; it referred to “commissioning and testing”, which subsequently led to the first validation guidance for clean rooms and septic processes. After that, the approach to validation became more sophisticated, and the volume of documentation increased. The main changes relate to technological improvements for a better understanding of processes and risks to the product (13).

The development of different regulations per country required the separate, prior evaluation of each new drug for commercial use by the health authorities of each country, respectively. This could be a very lengthy and drawn-out process. However, in the context of globalization, drug registration has evolved, allowing the rationalization and harmonization of requirements between countries to determine the safety, efficacy, and quality of the same drug. Europe led the need to rationalize and harmonize national regulations in 1980 when the development of a single market for pharmaceutical products began. Shortly after, a dialogue was established between Europe, the United States, and Japan on the possibility of harmonizing the requirements between the three regions. In 1990, as a result of this dialogue, the International Conference on Harmonization (ICH) for analyzing the technical specifications for registering medicinal products for human use was established. As a joint project of regulatory authorities and the pharmaceutical industry, they sought to identify divergent issues between work methodologies. The chosen topics encompass the criteria of authorization, quality, safety, and efficacy. The first conference took place in 1991, and conferences continue to be held approximately every 2 years (14).

## Comparison of international regulation

Worldwide, the principal GMP regulations are the EU Guide, the FDA Code of Federal Regulations (21 CFR Parts 210, 211, 610), and WHO GMP Guide, which have minor differences. Therefore, it becomes relevant to study these differences regarding requirements and production costs (15).

The first chapter of the EU Guide (“Pharmaceutical Quality System”) is divided into Quality Assurance, Good Manufacturing Practice for Medicinal Product (GMP), Quality Control, Product Quality Review, and Quality Risk Management. The WHO specified the second section as the main principles for pharmaceutical products, and also in Annex 3 describes Quality management in the medicines industry: philosophy and essential elements. However, the contents and requirements are largely the same (16).

In terms of concepts, the EU guide speaks of “the holder of Manufacturing Authorization” and “medicinal product” instead of “the manufacturer” and “pharmaceutical products”.

### Starting materials and manufacturing process

The WHO specifies that job descriptions must fix responsibilities in the first section. The controls are necessary for starting materials, bulk products, and calibration equipment. The GMP requirements are identical except for minor deviations. For example, the WHO refers specifically to additional cross-contamination and mix-ups as a risk for pharmaceutical production. In the EU guidelines, the critical steps and significant changes must be validated (referring to processes and analytical methods) rather than qualified (17). In the EU, the term “qualification” is used more specifically for equipment, utilities, and computer systems. The EU and the WHO regulations discuss periodic quality reviews, starting material, batches produced, critical in-process-control results, and finished products.

For good manufacturing practices, it is fundamental to have adequate facility construction and appropriate equipment suitable for the intended work tasks, to minimize the failure risk and to be easy to clean and maintain to avoid cross-contamination, which would affect product quality (18, 19). The prerequisite for vaccine areas is production, weighing, quality control, manufacturing, washing, cleaning, storage, sterilization, and maintenance of sterility.

Generally, it requires sufficient trained and qualified personnel to establish and maintain an effective quality management system in drug production (18, 20).

Good documentation is linked to the implementation of a GMP system as a basic requirement for the production of medicinal products of a high-quality level. It has to be intelligible and detailed and specify the process's instructions and records. For example, the specifications, master formulae, packaging instructions, batch processing records, batch packaging records, procedures or standard operating procedures and records, cleaning and sanitation are some essential documents. These documentation requirements can be found in chapter 15 of the WHO (16) and chapter 4 of the EU guideline (21), specifying the application of the regulations.

In order to produce a high-quality pharmaceutical product, the production process must be detailed to prevent cross-contamination during production, with the appropriate starting, intermediate, and bulk products, packaging materials, and finished products, with a continuous validation of the process. The management of distribution, rejected, recorded, and returned products are included in the validation (20, 22). There is a specific Annex that explains the Validation process in each guide. However, unlike the EU guideline, the WHO regulations indicate the time limit for the equipment storage after cleaning.

### Quality control

The quality of the product and the process is validated by a quality control department that covers sampling, starting specifications, execution of tests, as well as organization and documentation of release methods. In addition, testing is required for starting and packaging material, in-process control, finished products, and batch record review and retention samples (16, 23). The WHO specifies an independent department for Quality Control, including qualification and validation (16), and the EU defines an adequate facility for analysis (17).

Only the WHO guidelines include storage as a process that has to be monitored to minimize risks to product quality.

Some other topics covered include contract manufacture and analysis, complaints and product recall, and self-inspection, which are similar in both guides (16, 17).

### Heating, ventilation, and air-conditioning system

Finally, an important technical support area for a pharmaceutical product is the HVAC system for pharmaceutical dosage forms. It has to provide comfortable work conditions for the operational staff and prevent any climatic change that could negatively affect the manufacturing process. According to Annex 8 of the WHO (24), preventing contamination and cross-contamination is an essential design consideration of the HVAC system.

WHO has an entire chapter for HVAC systems specific for non-sterile pharmaceutical dosage forms and gives information on sterile production (25, 26). However, the pharmaceutical industries that follow the EU regulation have limited access. For example, one of the guidelines is the Handbook of the non-profit technical organization “American Society of Heating, Refrigerating and Air-Conditioning Engineers, Inc.”, which is the main document considered for the HVAC system regulation (27). Another important standard for HVAC systems is the ISO 14644 series of standards, which define Airborne Particulate Cleanliness Classes in Cleanrooms and Clean Zones (28). This document is an engineering design standard frequently referenced in EU and FDA guidelines. In the design, airborne particulate cleanliness and control of clean rooms and environments must be considered. These considerations include air classification, the standards defined for the particle concentrations, and the permissible microbial concentrations.

The validation and qualification processes are essential parts of modern GMP practice because the validation process proves that any procedure, process, equipment, material, activity, or system leads to the expected results, and qualification demonstrates that any premises, system, and equipment work correctly. These activities include validating HVAC systems, water systems for pharmaceutical use, cleaning processes, analytical methods, computer systems, equipment, and non-sterile processes. Each guide defines a specific validation process that has to be followed step by step.

### Pharmaceutical product

In order to achieve a good quality of the finished product, the EU GMP guideline and the WHO define rules and recommendations for producing active pharmaceutical ingredients, so-called APIs. In both guidelines, the normative requirements are similar, with some differences in chapters 1 and 2 of the WHO, which included sections 1.2, “Regulatory applicability”, and 1.3, “Scope” (27).

### Staff responsibilities

In order to avoid cross-contamination, the respective responsibilities must be clearly defined, and staff must receive training in individual hygiene practices. In addition, concepts and responsibilities are described, such as those of the “qualified person,” “authorized person,” “Head of the Production Department,” or “Head of Quality Control.” The WHO stresses, as well as EU and FDAs guidance, specified the necessity of a Quality Management System in the areas of production and control (18) of pharmaceutical products and active ingredients. Quality Management includes all the organizations to ensure the quality required for the intended use. In the same way, the WHO guidelines specify the necessity of equipment calibration and validation as part of quality control.

## GMP monoclonal antibody and vaccine production strategy

Monoclonal antibodies and vaccines are biological products, sterile manufactured (29). The production process of these products is the most challenging and risky in the industry. Biological products are derived from cells, tissues, or microorganisms and reflect the inherent variability characteristic of living materials. The active substances are difficult to characterize by utilizing physicochemical testing methods because they may show heterogeneity between one lot to another. Consequently, special considerations are needed to maintain consistency in product quality (7). Biological products can be obtained from processes such as the cultivation of strains of microorganisms and eukaryotic cells; extraction of substances from biological tissues, including human, animal, and plant tissues and fungi; recombinant DNA techniques; hybridoma techniques; and propagation of microorganisms in embryos or animals. Vaccines are produced by culturing strains of microorganisms or DNA techniques, and monoclonal antibodies are obtained by hybridoma techniques (7).

The EU and WHO guides (29, 30) establish rules to minimize the risk of microbiological contamination and implement sterilization processes. It focuses on the ISO 14644-1 (28) standard air for classification. In addition, environmental monitoring, bioburden testing, media fills, and 100% integrity testing of container closures are essential in the sterile process. These tests should be repeated at defined intervals and after significant modification to the HVAC system, equipment, or process (31).

Critical steps, such as the final sterile filtration, aseptic filling process, and capping vials, must be performed under grade A laminar flow within a grade B environment or in a grade A room. Therefore, the grade A area is the most critical, which must be continuously monitored during all operations. Also, in grade A rooms, a unidirectional flow with a homogeneous airspeed of 0.36–0.54 m/s is required (9), and environmental conditions must be demonstrated and validated by undertaking airflow visualization tests.

Additionally, to determine the sterility of the process, it is necessary to do bioburden testing before the final sterilization of the product, generally by filtration for biological products such as vaccines and monoclonal antibodies. This involves estimating the number of aerobic microorganisms such as bacteria and fungi in the sample, commonly assessed using the Total Viable Count (TVC) method, as a limit of 10 CFU/100 mL (32). To determine growth, the methods are membrane filtration, pour plate, and spread plate, and the results are reported by colony forming units (CFU). After the incubation of the sample under the appropriate conditions, the CFU is typically zero. This ensures that the final filtration or heat sterilization challenge is minimal, providing a greater probability of sterility (33). The working conditions in which the test is performed are monitored regularly by carrying out controls and appropriate sampling of the working area.

A process simulation test using a nutrient medium corresponds to the media fill test, where the nutrient medium should be selected based on selectivity, clarity, concentration, and suitability for sterilization of the nutrient medium (31). This simulation test should imitate as closely as possible the aseptic manufacturing steps and should be performed by running three consecutive satisfactory simulation tests (31).

As previously mentioned, the HVAC system plays an important role in preventing contamination and cross-contamination and needs to be considered at the initial design stage of a pharmaceutical manufacturing plant, with permanent control and monitoring to ensure compliance with the operating parameters. These parameters extend to particulate, temperature, humidity, and pressure design. To remove contaminants, HEPA filters must be installed; when necessary, a competent person should change them. In addition, filter integrity (leakage) testing must be carried out periodically (23). The frequency of this integrity test can vary widely in different regions. For example, the EU recommends grade A & B filters every 6 months and 12 months for grade C & D filters (34). Finally, microbiological environmental monitoring ensures minimal microbiological risks during vaccine dispensing.

Furthermore, as was mentioned in the previous section, the control of clean rooms and environments must be considered (28). Finally, it is necessary to count and size the airborne microparticles and compare them with the maximum permitted concentration. There are two categories of microparticle measurement methods, the first one is *in situ* measurement, and the other is the collection by filtration or inertial effects, followed by microscopic measurement of the number and size of collected particles (28).

## Impact of GMP plant and production on public health

The effects of COVID-19 have profoundly impacted drug use and public health spending. Global spending on medicines, including COVID-19 vaccines and therapeutics, reached US$1,521 billion in 2021 and is expected to be US$1,805 billion in 2026 (35). In Chile, in February 2021, the vaccination campaign against COVID-19 began, and more than 57 million doses have been administered since that date. Table 1 shows statistics of the number of doses administered until July 01, 2022, according to the vaccination schedule (36). During the pandemic, the importance of vaccine and antibody development became apparent. This was reflected in a considerable increase in the demand for GMP laboratories for the production and scaling up of new technologies under development, which for many companies made significant investments to expand production capacities that finally allowed the supply of a large number of medicines for human use (36).

**Table 1:** SARS-CoV-2 vaccination campaign, in Chile until 01 July 2022.

	**Over 18 years old**		Under 18 years old*	
**Target population**	**15.200.840**		**3.771.760**	
**Vaccination scheme**	**Vaccinated people**	**% Target population**	**Vaccinated people**	**% Target population**
Complete scheme	14.280.556	93,9	3.290.566	87,2
First booster	13.094.008	86,1	1.916.045	50,8
Second booster	9.213.145	60,6	192.824	5,1
Total administrated doses	50.506.551	-	8.709.578	-

Department of Health Statistics and Information, Ministry of Health, Chile. Report updated Friday, July 01, 2022, 13:12:05 at previous day data (36).

Under 18 years old scheme, vaccination is currently in progress because the vaccination strategy was applied from older to younger ages.

Different tests must be performed in the drug research and development process to ensure that the new products present toxicity, safety, immunogenicity, and efficacy parameters within the regulatory entity's standards (17–23, 37). To carry out these tests in their human clinical studies, they must use formulations produced under good manufacturing practices; otherwise, in addition to the health risks, it also implies an increase in the cost of the pharmaceutical product for both governments and customers (38). Furthermore, unlike other products, it is challenging for a customer to identify (through taste, smell, or appearance) whether the medicine is in good condition, is of good quality, or works properly, which is why it is imperative to have all the controls that guarantee these parameters (39).

Once the regulatory bodies have approved the drugs after performing all the required studies and trials, they must be scaled up to mass production to supply the market demand (37). This requires GMP facilities that allow large-scale production of the approved drug formulations, with the necessary personnel, facilities, certified equipment, supply of raw materials, and qualified personnel. This represents a significant challenge since large volumes must be produced while maintaining excellent sanitary and quality conditions throughout the manufacture of the product.

Vaccine manufacturing facilities are specific and expensive for large-scale production and quality standards to ensure consistency and control. Therefore, most countries have contract agreements with specific cGMP-certified manufacturers to purchase the vaccines for their population. For example, the European public and private sectors provide vaccines, allowing the public health system to access different sources and prices (40). The leading pharmaceutical companies that produce and distribute vaccines around the world include GSK (United Kingdom); Pfizer (USA); Sanofi Pasteur (France); Merk & Co. (USA); Roche (France); Seqirus (Australia); Valvena SE (France); Sinovac (China); Sinopharm (China). In addition, some emerging companies such as Astellas Pharma and Takena in Japan; AstraZeneca in the United Kingdom. Furthermore, the Serum Institute of India is a state-owned vaccine manufacturing center that produces most of the vaccines recommended by the WHO, such as BCG, Polio, Hib, DTaP, and MMR. This institute, in particular, supplies vaccines to Chile, which does not have a vaccine manufacturing facility (10).

In Latin America, facilities in Argentina, such as Sinergium Biotech, can produce vaccines for Influenza, Pneumococcus, and HPV, and ANLIS, which makes BCG, Rabies, Tetanus Toxoid, and Yellow Fever vaccines. Similarly, two national public enterprises in Brazil, Immunobiological Technology Guinhos (Bio-Manguinhos/Fiocruz) and Butantan Institute can supply most vaccines for the Brazilian population (41).

**Figure 2:**
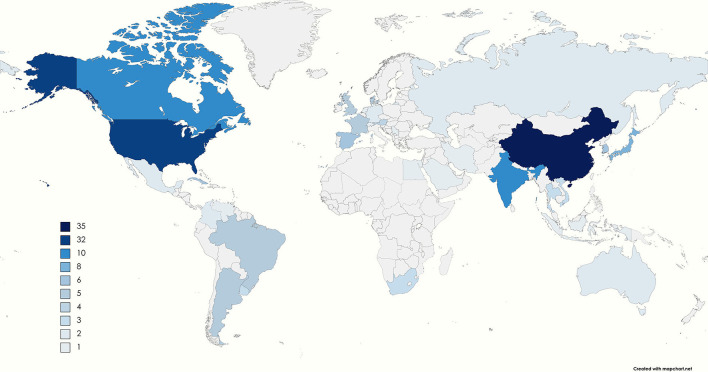
The number of GMP manufacturing companies in each country (41–104). The color range goes from light blue to dark blue, and the gray sections indicate that no companies are governed under the GMP requirements. The legend shows the number of GMP manufacturers in each country. The countries with only one GMP industry are Egypt, Saudi Arabia, Indonesia, Irán, Serbia, Netherlands, Switzerland, and Venezuela. In contrast, the country with the most is China, with 35, followed by the United States with 32 industries.

On the other hand, South Korea, China, Thailand, Indonesia, Vietnam, Iran, Egypt, Cuba, and Mexico produce, store and distribute vaccines in public immunization programs. In addition, some countries have new facilities, such as Brazil with Bio-Manguinho Institute, the Russian Federation with Gamaleya National Research Center, India with the Serum Institute of India, China with Sinovac, and South Africa with Aspen Pharmacare (40). Figure 2 shows the distribution of GMP industries worldwide, where some are producers of vaccines and others of other pharmaceutical products. The color reference indicates the number of industries in each country. China is the country with the largest number of facilities, followed by the United States. In South America, there are 16 plants, mainly concentrated in Argentina and Brazil. Table 2 shows the predominant industries in each country.

**Table 2:** List of leading GMP manufacturing facilities worldwide (41–104).

**Country**	**Main GMP manufacturing companies**
China	Sinovac Biotech Ltd, Cansino Biologics Ltd., China National Biotech Group
Japan	Shionogi & Co, Takeda Pharmaceutical Company, KM Biologics Co., Ltd
Thailand	Siam Bioscience Co, Government Pharmaceutical Organization, BioNet.
Vietnam	VABIOTECH, Institute for Vaccines and Medical Biologicals, Nanogen Pharmaceutical Biotechnology.
Egypt	Vacsera
South Korea	SK Bioscience, Green Cross, Hanmi Pharmaceutical Co.
Saudi Arabia	SaudiVax
India	Serum Institute of India, Bharat Biotech International Ltd, Zydus Cadila
Indonesia	Bio Farma
Irán	Razi
Serbia	Torlak Institute
Russia	Petrovax Pharm, Gamaleya Research Institute of Epidemiology and Microbiology
Netherlands	Batavia Biosciences, Wacker Biotech
France	Sanofi Pasteur, Laboratoires Servier, Viatris Santé
Germany	BioNTech, CureVac
Italy	Laboratory of Cell and Gene Therapy Stefano Verri, Areta International Srl, MolMed Spa
Spain	MSD, Zendal Groups, Algenex—Biotech Spain
UK	Albumedix, GSK, Oxford Vaccine Group
Austria	Vienna BioCenter, Themis Bioscience, Nuvonis
Denmark	AJ Vaccines, Bavarian Nordic, AdaptVac
Canada	Medicago, BIOTECanada, Biologics Manufacturing Center
Switzerland	Novartis
South Africa	The Biovac Institute, Aspen Pharmacare
Australia	Seqirus, University of Queensland
United States	Merck & Co., Inc., Johnson & Johnson, Pfizer
Brazil	Bio-Manguinhos/FioCruz, Butatan Institute San Paolo, Technological Institute of Parana
Mexico	Birmex, National Institute of Virology
Colombia	VaxThera, The National Institute of Health, FIDIC
Cuba	Finlay Institute, CIGB, BIOCEN
Venezuela	National Institute of Hygiene Rafael Rangel
Uruguay	Pasteur Institute of Montevideo, Clausen Laboratory, GBT Grupo Biotoscana
Argentina	Sinergium Biotech, Malbrán Institute, INEVH
Chile	IMII—Pontificia Universidad Católica de Chile, Centro de Producción de Vacunas y Biofarmacéuticos del Parque Carén, Sinovac Biotech Chile (Under construction)

MSD, Merck Sharp & Dohme; GSK, GlaxoSmithKline; FIDIC, Foundation Institute of Immunology of Colombia; CIGB, Center of Genetic Engineering and Biotechnology; BIOCEN, National Bio-preparations Center; INEVH, National Institute of Human Viral Diseases Julio Maiztegui; IMII, Instituto Milenio en Inmunología e Inmunoterapia.

### Impact of cGMP processes on vaccines manufacture

As mentioned above, the entire vaccine manufacturing process must follow GMP standards, thus meeting all the necessary asepsis requirements. In the development of a vaccine, there are different standardized stages. First is the experimental phase, followed by preclinical testing and clinical development, which consists of three phases. Finally, prior to marketing, it must be approved and pharmacovigilance.

The COVID-19 pandemic highlighted the importance of having regulations in place to ensure product asepsis for the entire process of vaccine research and development. The guidelines provided by the different regulatory agencies made it possible to have vaccine prototypes that could be evaluated for tolerance, safety, immunogenicity, and efficacy in the different clinical stages of pharmacological development, to scale up production volumes in manufacturing plants that were fully GMP certified, and also enabled the manufacture of doses for the human population in the world. Even in the preclinical phase, in order to mitigate risk, sometimes the doses supplied are formulated under current Good Manufacturing Practices, and tests are performed to determine the immunogenicity, tolerance, safety, and toxicity of the product in cell cultures, tissues, and animal models, such as monkeys, mice, and rats.

Examples of vaccines produced under GMP regulations include those based on the innovative technique using recombinant Bacillus Calmette-Guerin (BCG), a live attenuated vaccine widely used since 1921 to prevent disease caused by Mycobacterium tuberculosis (97). This recombinant platform is capable of expressing viral particles that confer immunogenic protection against viruses such as a human respiratory syncytial virus (hRSV), human metapneumovirus (hMPV) (103–106), Andean ortho-hantavirus (ANDV), and SARS-CoV-2, the pathogen responsible for coronavirus disease 2019 (103–118).

The hRSV is the most important cause of hospitalizations in children under 2 years of age for lower respiratory tract infection, and there is currently no commercially available vaccine against this disease (104, 105, 118). We have shown that a recombinant BCG expressing the hRSV nucleoprotein (rBCG-N-hRSV), produced following cGMP standards, promotes virus clearance and prevents lung damage in vaccinated mice (104, 105). Similarly, a single, low dose of rBCG expressing the hMPV phosphoprotein was shown to provide a protective and balanced immune response in mice against hMPV disease (119). Furthermore, following preclinical trials, the clinical development of the rBCG-N-hRSV vaccine was initiated by conducting a phase 1, double-blind, dose-escalating clinical trial in healthy males aged 18–50. It was concluded that the rBCG-N-hRSV vaccine produced following cGMP standards was well tolerated and safe, and no serious vaccine-related adverse events were reported (119).

Similarly, under GMP requirements, BCG was used to develop a recombinant vaccine expressing the SARS-CoV-2 nucleoprotein to evaluate immunogenicity and safety by immunizing mice, where they concluded that immunization with two doses of 1 × 10^8^ CFU or one dose of 1 × 10^5^ CFU of these BCG was safe, and a significant cellular immune response was induced (120). Under the same standards in Chile, a Phase 3 clinical study of an inactivated vaccine against SARS-CoV-2 was conducted with the CoronaVac vaccine produced following cGMP requirements at Sinovac Biotech Ltd. The safety and immunogenicity of a subgroup of healthy adults (121), the recognition of the variants of interest by antibodies and T cells (122), and the immune profile and clinical outcome of breakthrough cases after vaccination with CoronaVac were evaluated (122). After analyzing all the results, it has been determined that immunization with CoronaVac is safe and induces an immune response in healthy adults, that it promotes the secretion of antibodies capable of blocking the SARS-CoV-2 variants of interest and partially neutralizes SARS-CoV-2 infection and that the breakthrough cases mainly were mild and did not necessarily correlate with a lack of vaccine-induced immunity (120–122), allowing regulatory approval of the vaccine and consequently the vaccination of millions of people in Chile.

None of these vaccines could have reached pre-clinical or clinical stages without the support of GMP production facilities capable of manufacturing the vaccines at the appropriate scale. Thus, demonstrating the importance of GMP standards in vaccine production, which was reaffirmed following the 2019 coronavirus disease pandemic. Also, due to good results in preclinical studies of recombinant BCG vaccines and intending to be able to produce this type of vaccine in one place, the Institute of Immunology and Immunotherapy is building a GMP rBCG vaccines plant in Chile.

## Conclusions

To meet essential quality standards of a pharmaceutical product, it is necessary to comply with GMP regulations. Worldwide, countries generate their own GMP regulations based on WHO recommendations. From these recommendations, the European and PICS regulations have evolved, and all these GMP regulations include the qualifications and training of the staff, sourcing of raw materials, in-process and release quality control, production equipment and production methods, storage of intermediates, and final product, record keeping, and quality assurance. In Chile, the Public Health Institute regulates the production of pharmaceutical products and approves the installation and operation of this type of facility. The Chilean standard is based on the WHO regulation with some changes that are specific to the country.

Also, as it was mentioned that the highest quality standards must be met, many countries do not have their own GMP manufacturing industries, and, on the contrary, China and the United States are the countries with the most GMP companies.

Due to the increased scale in the use of vaccines and the emergence of new technologies, regulations have become stricter for producing these products due to the increased risk involved. This increase in regulation has given rise to problems in production, loss of the GMP in the manufacturing centers, and changes in the formulations, which explain the disruptions of vaccine supplies. Also, because fewer vaccine manufacturing suppliers exist for a given vaccine, the impact on the supply shortage of a growing population has increased. Therefore, the challenge for governments and institutions is to invest in local vaccine development and manufacturing to avoid foreign dependency and the threat of shortage. This would have a significant impact on public health in Latin American countries.

## Author contributions

All authors listed have made a substantial, direct, and intellectual contribution to the work and approved it for publication.

## Funding

CORFO (13CTI21526–P4), Millennium Institute on Immunology and Immunotherapy (ICN09_016/ICN 2021_045), ANID (FONDECYT 1190830). This study received funding from Millennium Institute on Immunology and Immunotherapy, a non-profit institution. The funder was not involved in the study design, collection, analysis, interpretation of data, the writing of this article or the decision to submit it for publication.

## Conflict of interest

Author TD was employed by the company Deeks Pharmaceutical Consulting Services. The remaining authors declare that the research was conducted in the absence of any commercial or financial relationships that could be construed as a potential conflict of interest.

## Publisher's note

All claims expressed in this article are solely those of the authors and do not necessarily represent those of their affiliated organizations, or those of the publisher, the editors and the reviewers. Any product that may be evaluated in this article, or claim that may be made by its manufacturer, is not guaranteed or endorsed by the publisher.
